# Supplementary material for: Target residence of Cas9-sgRNA influences DNA double-strand break repair pathway choices in CRISPR/Cas9 genome editing
Source: Genome Biol. 2022 Aug 1;23:165. doi: 10.1186/s13059-022-02736-5 (PMC9341079; doi:10.1186/s13059-022-02736-5)
Supplement: Supplementary file 1 — Additional file 1: Figure S1. Generation of DNA-PKcs–/– and Ku80–/– HDR reporter mESC clones. Figure S2. The effect of DNA-PKcs inhibition on the patterns of Cas9-induced NHEJ analyzed by targeted PCR amplicon Illumina sequencing. Figure S3. The effect of DNA-PKcs inhibition on the patterns of Cas9-induced NHEJ at the Cola1 and Rosa26 locus. Figure S4. Effect of DNA-PKcs inactivation on I-SceI-induced mutNHEJ. Figure S5. Sequences of mismatched or truncated sgRNAs used for weakening target interaction of Cas9-sgRNA. Figure S6. Effect of DNA-PKcs inhibition on SpCas9-mediated gene knockout (KO) at GFP gene. Figure S7. Replication locally disengages c-NHEJ at Cas9-induced DSBs. Figure S8. Analysis of palindromic sister chromatid ligation. Figure S9. Junctions of palindromic sister chromatid NHEJ products. [file 13059_2022_2736_MOESM1_ESM.pdf]

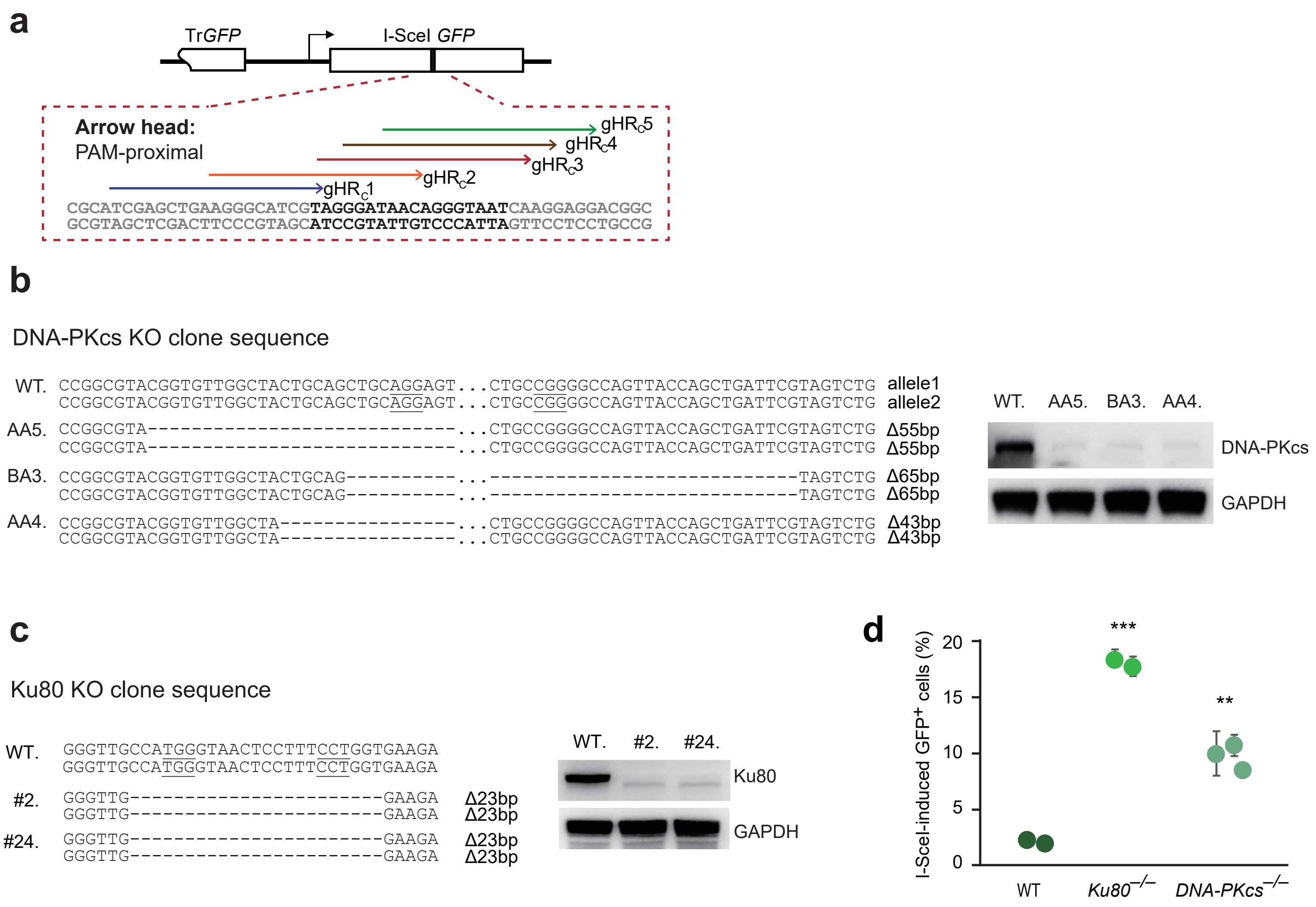

Fig S1 Generation of *DNA-PKcs*$^{-/-}$ and *Ku80*$^{-/-}$ HDR reporter mESC clones. **a** Schematic of the orientations and distances of the tandem Cas9 target sites at the HDR reporter. **b,c** HDR reporter mESC were transiently transfected with expression plasmids for paired SpCas9-sgRNAs respectively targeting exon 1 of *DNA-PKcs* (**b**) and exon 2 of *Ku80* (**c**), and the plated on MEFs. In about 2 weeks, thee *DNA-PKcs* $^{-/-}$ and two *Ku80* $^{-/-}$ HDR reporter mESC clones along with two isogenic wild-type (WT) clones were identified by Sanger sequencing and Western blot. **d** Clones were transfected with I-SceI expression plasmid and frequencies of I-SceI-induced HDR represented by percentages of I-SceI-induced *GFP*$^{+}$ cells. Each circle indicates the mean ± S.E.M. of three independent experiments, each in triplicates, with individual clone. Significance was detected by two-tailed Student's unpaired t-test and indicated by ** for P<0.01 and *** for P<0.001.

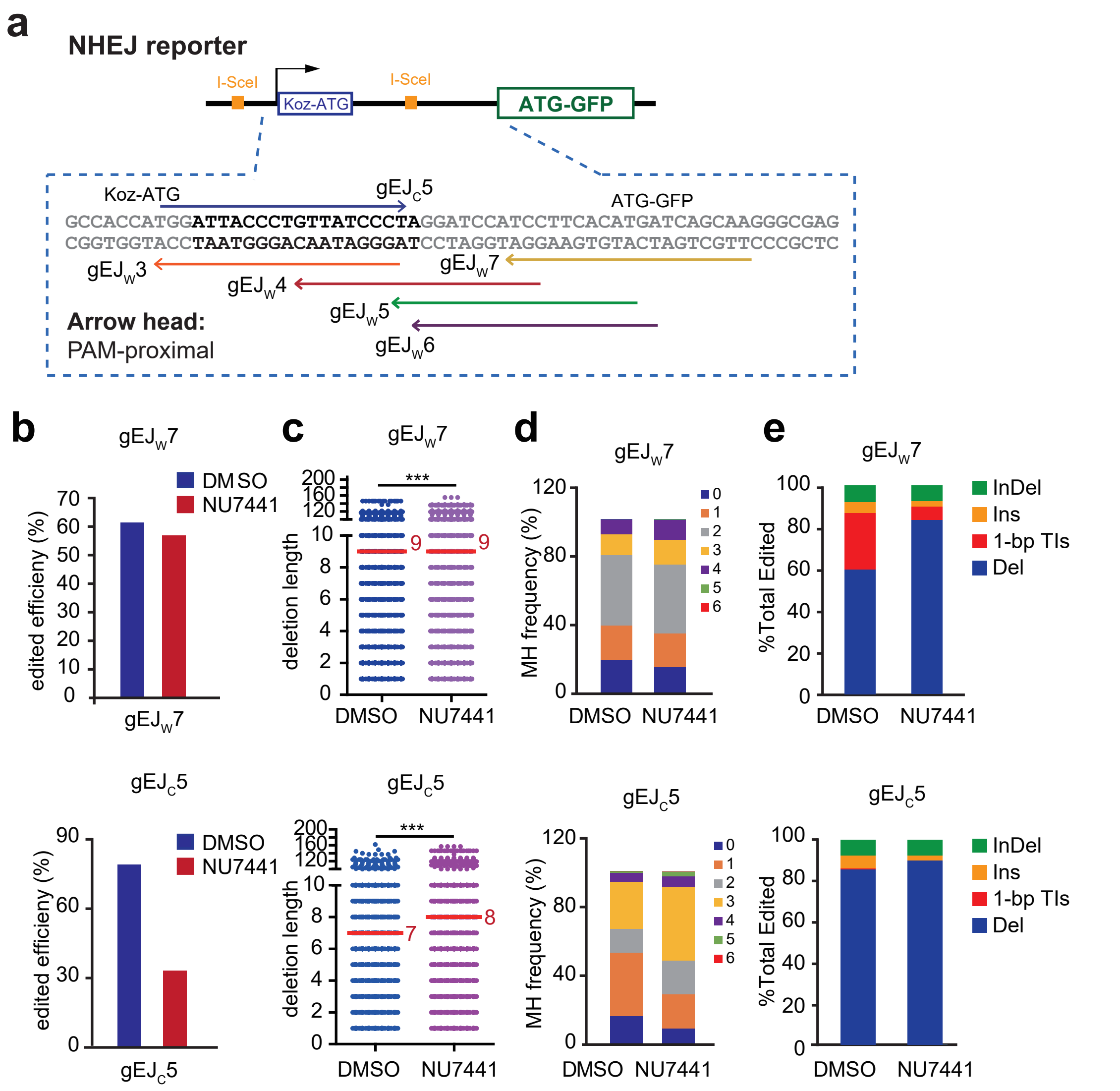

Fig S2 The effect of DNA-PKcs inhibition on the patterns of Cas9-induced NHEJ analyzed by targeted PCR amplicon Illumina sequencing. **a** Schematic of the orientations and distances of the Cas9 target sites at the NHEJ reporter. **b-e** The pattern of Cas9-induced NHEJ from mESC treated with DMSO or NU7441 at gEJ$_W$7 (**top**) and gEJ$_C$5 (**bottom**) sites. The edited efficiency was calculated as the ratio of edited reads to total reads from targeted Illumina sequencing and normalized by transfection efficiency (**b**). The length distribution of deletions with median deletion length is indicated (**c**). Each dot represents 100 reads. The frequency of MH and the number of homologous bases were grouped as indicated (**d**). The frequencies of deletion (Del), insertion including 1-bp templated insertion (TIs) and the other insertion (Ins), and InDel were calculated as the percentage of total edited (**e**). Deletion distributions between DMSO and NU7441 in **c** were compared by a two-tailed Mann–Whitney test. ***, P < 0.001.

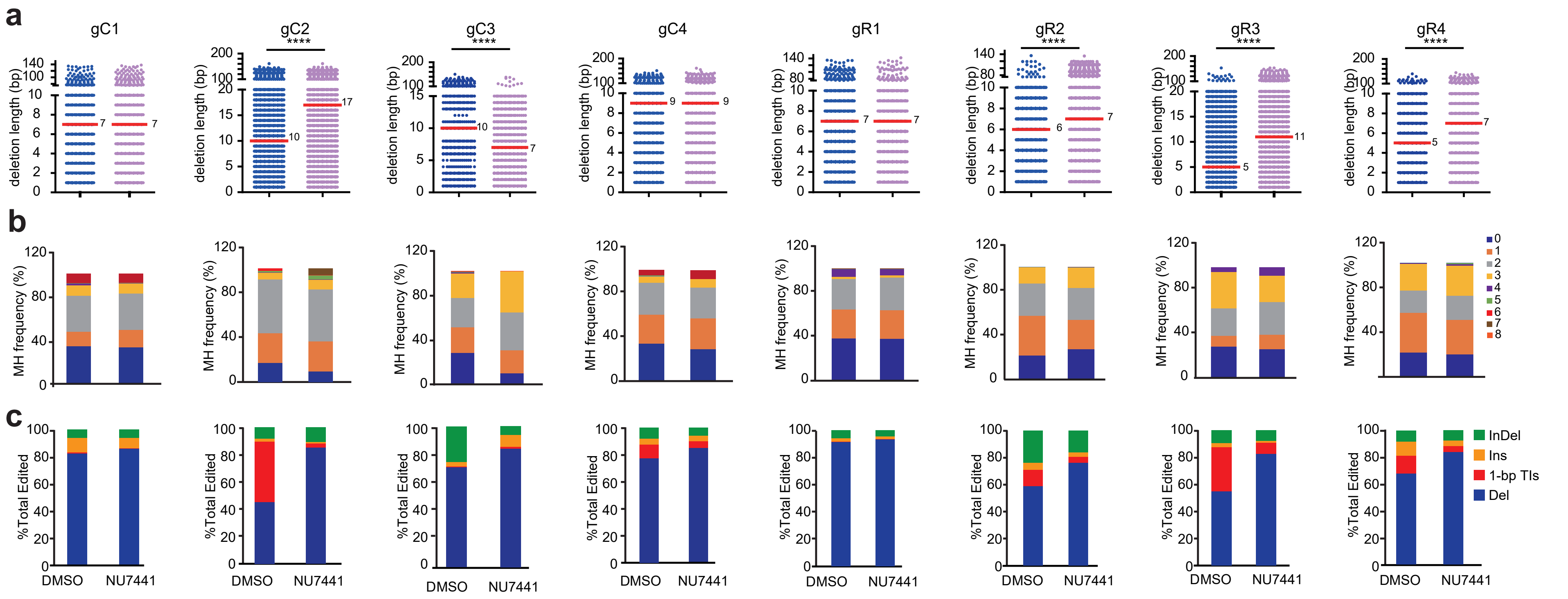

Fig S3 The effect of DNA-PKcs inhibition on the patterns of Cas9-induced NHEJ at the *Cola1* and *Rosa26* locus. **a** The length distribution of deletions with median deletion length is indicated. Each dot represents 100 reads. Deletion distributions between DMSO and NU7441 were compared by a two-tailed Mann–Whitney test. ****, P < 0.0001. **b** The frequencies of MH and the number of homologous bases were grouped as indicated. **c** The frequencies of deletion (Del), insertion including 1-bp templated insertion (TIs) and the other insertion (Ins), and InDel were calculated as the percentages of total edited.

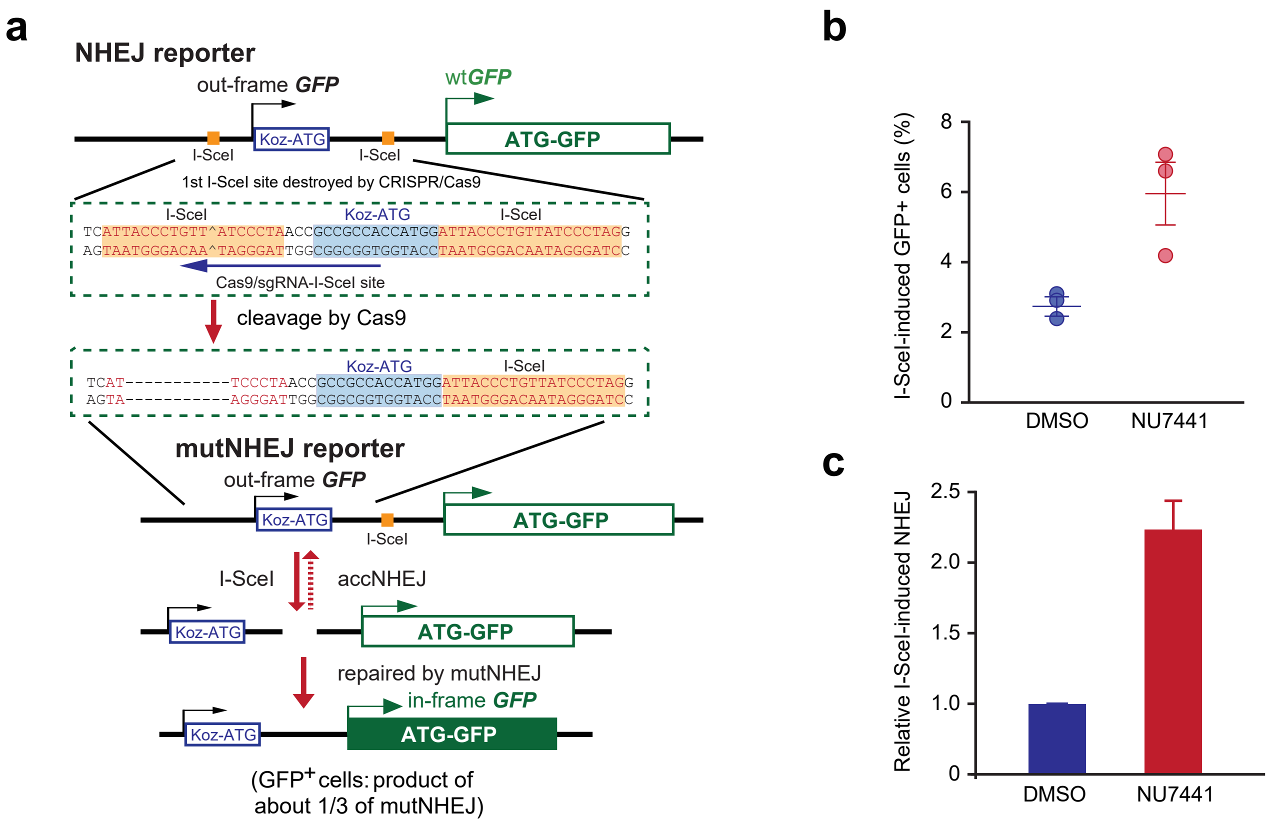

Fig S4 Effect of DNA-PKcs inactivation on I-SceI-induced mutNHEJ. **a** Generation of the mutNHEJ reporter. The first I-SceI site in the original NHEJ reporter was disrupted by CRISPR/Cas9 genome editing as shown, generating the mutNHEJ reporter that contains only a single intact I-SceI site. A site-specific DSB can be induced between Koz-ATG and *ATG-GFP* and subsequent repair of the DSB by mutNHEJ has 1/3 probability in theory to correct out-of-frame *GFP*, generating *GFP* cells. Dotted box indicates the targeting and editing of the first I-SceI site in the NHEJ reporter by Cas9-sgRNA. Sanger sequencing confirms the disruption of the first I-SceI recognition sequence. **b,c** Effect of DNA-PKcs inhibition on I-SceI-induced NHEJ in mutNHEJ reporter mESC transfected with I-SceI expression plasmids. Frequencies of I-SceI-induced *GFP* cells (**b**) were measured by FACS, and relative I-SceI-induced NHEJ was calculated by normalizing DMSO treatment to 1.0 (**c**). Each circle indicates one independent experiment, each in triplicates, and the mean of three independent experiments is also indicated. Error bars indicate S.E.M. Columns indicate the mean ± S.E.M. of relative NHEJ. Two-tailed Student's paired t-test: ** for P<0.01 and *** for P<0.001.

a sgRNAs and their variants for NHEJ reporter targets

gEJ$_c$5 target (20 … 1) TGGATTACCCTGTTATCCCT AGG (PAM)

gEJ$_c$5 and variants

gEJ$_c$5	TGGATTACCCTGTTATCCCT
C2A	TGGATTACCCTGTTATCCAT
T15A	TGGATAACCCTGTTATCCCT
16nt	TTACCCTGTTATCCCT

gEJ$_W$7 target (20 … 1) CTTGCTGATCATGTGAAGGA TGG (PAM)

gEJ$_W$7 and variants

gEJ$_W$7	CTTGCTGATCATGTGAAGGA
A1T	CTTGCTGATCATGTGAAGGT
A4C	CTTGCTGATCATGTGACGGA
T15A	CTTGCAGATCATGTGAAGGA

b sgRNAs and their variants for *mCola1* and *mRosa26* loci

gC4 target (20 … 1) GCCAGGAAGAGTCCCAAGAC TGG (PAM)

gC4 and variants

gC4	GCCAGGAAGAGTCCCAAGAC
G1T	GCCAGGAAGAGTCCCAAGAT
G16C	GCCACGAAGAGTCCCAAGAC

gR4 target (20 … 1) ACTGAGCTATTAACCATTAA TGG (PAM)

gR4 and variants

gR4	ACTGAGCTATTAACCATTAA
A1C	ACTGAGCTATTAACCATTAC
A16T	ACTGTGCTATTAACCATTAA

c sgRNAs and their variants for HDR reporter targets

gHR$_c$4 target (19 … 1) GATAACAGGGTAATCAAGG AGG (PAM)

gHR$_c$4 and variants

gHR$_c$4	GATAACAGGGTAATCAAGG
G1C	GATAACAGGGTAATCAAGC
G2C	GATAACAGGGTAATCAACG
17nt	TAACAGGGTAATCAAGG

gHR$_c$2 target (20 … 1) AGGGCATCGTAGGGATAACA GGG (PAM)

gHR$_c$2 and variants

gHR$_c$2	AGGGCATCGTAGGGATAACA
A1T	AGGGCATCGTAGGGATAACT
C2A	AGGGCATCGTAGGGATAAAA
17nt	GCATCGTAGGGATAACA

Fig S5 Sequences of mismatched or truncated sgRNAs used for weakening target interaction of Cas9-sgRNA targeting NHEJ reporter targets (**a**), natural genomic targets *mCola1* and *mRosa26* (**b**) and HDR reporter targets (**c**). Mismatched nucleotide in each sgRNA is indicated in red.

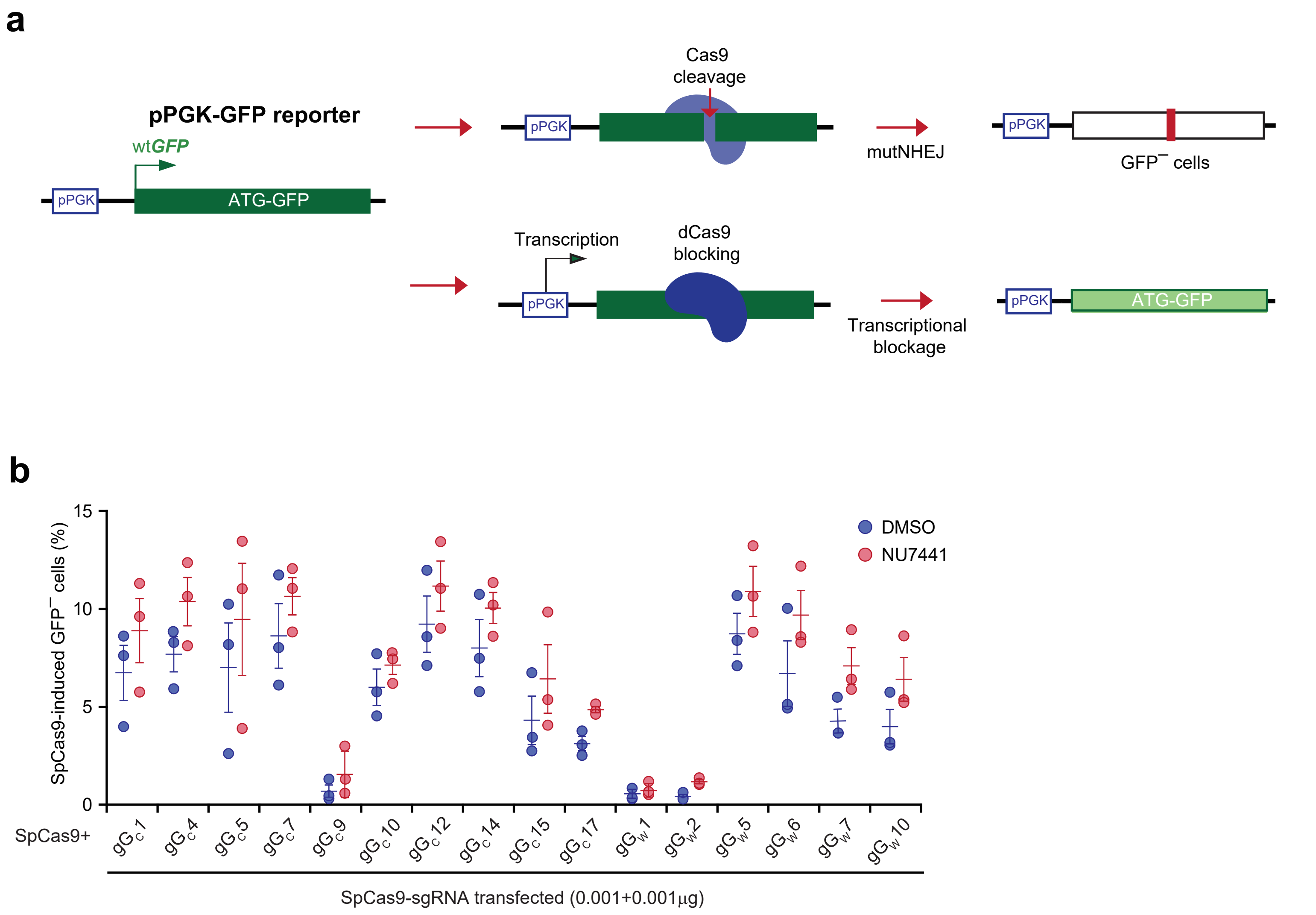

Fig S6 Effect of DNA-PKcs inhibition on SpCas9-mediated gene knockout (KO) at *GFP* gene. **a** Schematic of *GFP* gene KO and transcription blockage. A single copy of the *GFP* gene driven by the *PGK* promoter in mESC was targeted by SpCas9 in complex with sgRNAs designed. Upon site-specific DSB induction, DSB repair by m-NHEJ could inactivate GFP, making cells *GFP*$^{-}$. Frequencies of SpCas9-induced *GFP*$^{-}$ cells indicate the efficiencies of *GFP* KO. Targeting dSpCas9 to *GFP* can efficiently silence *GFP* expression. Frequencies of SpCas9-induced *GFP*$^{-}$ cells indicate the efficiencies of *GFP* transcription blockage. **b** mESC containing *pPGK-GFP* expression cassette were transfected with a low amount of individual SpCas9-sgRNA expression plasmids (0.001µg SpCas9, 0.001µg sgRNA, 1/500 of total DNA each) as shown and treated with NU7441 at 6 h post-transfection. Percentages of SpCas9-induced *GFP*$^{-}$ cells were measured by FACS at 4 d post-transfection and normalized by transfection efficiency. One circle indicates one independent experiment, each in triplicates, and the mean of these independent experiments is also indicated. Error bars indicate S.E.M. Relative SpCas9-induced NHEJ shown in **Fig 6b** is derived from these percentages of SpCas9-induced *GFP*$^{-}$ cells.

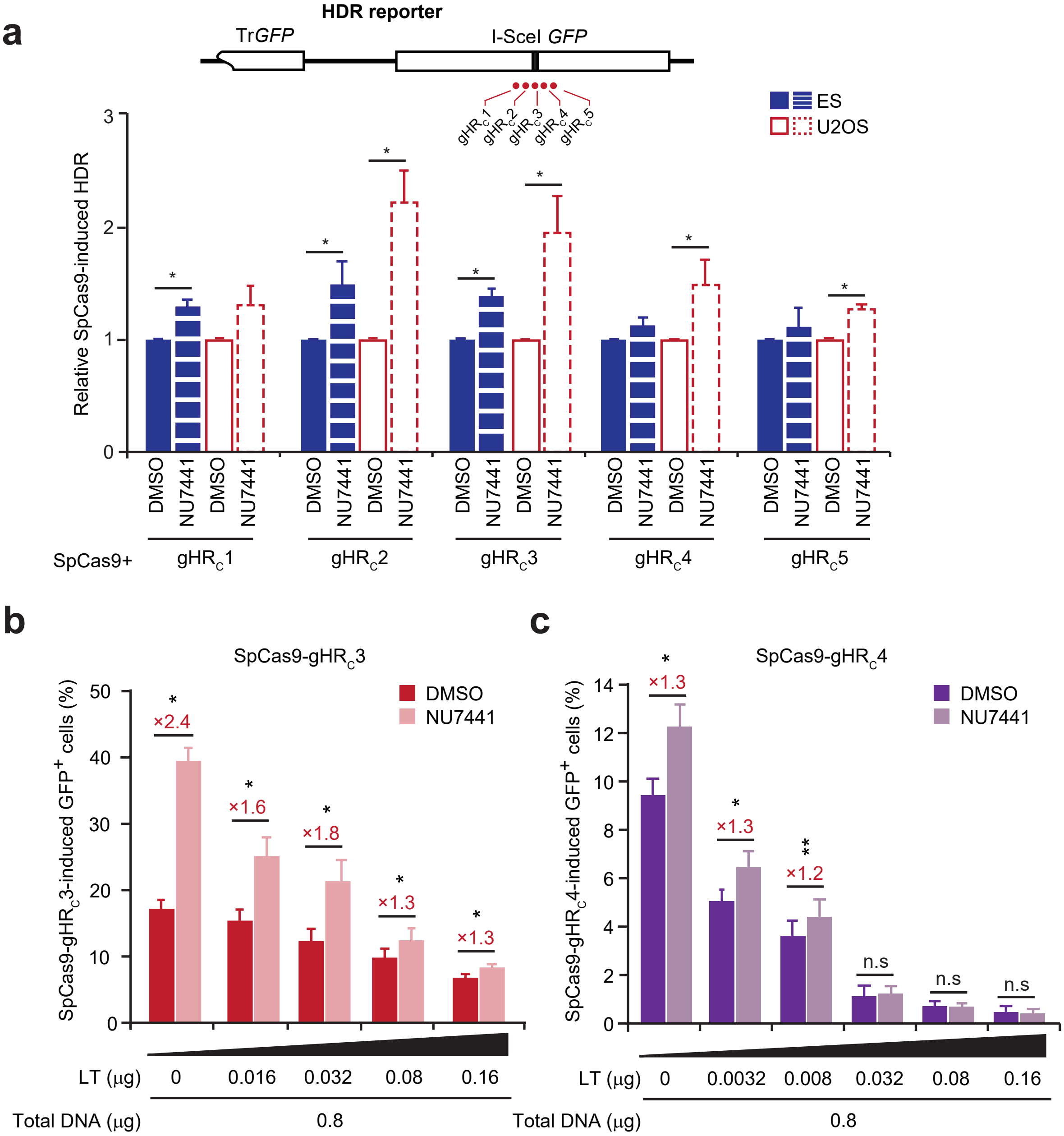

Fig S7 Replication locally disengages c-NHEJ at Cas9-induced DSBs. **a** Effect of DNA-PKcs inhibition on SpCas9-induced HDR in mESC and U2OS cells transfected with SpCas9-sgRNA expression plasmids as indicated and treated with either DMSO or NU7441. Relative SpCas9-induced HDR was calculated by normalizing DMSO treatment to 1.0 after frequencies of SpCas9-induced *GFP*$^+$ cells were measured by FACS at 3 d post-transfection. **b,c** Impact of local replication on DNA-PKcs involvement in HDR induced by SpCas9-gHRc3 (**b**) and SpCas9-gHRc4 (**c**). HDR reporter U2OS cells were transfected with SpCas9-sgRNA expression plasmids, together with different amounts of SV40 *LT* expression plasmids, and treated with either DMSO or NU7441. Frequencies of SpCas9-induced *GFP*$^+$ cells were measured by FACS at 3 d post-transfection. Columns indicate the mean ± S.E.M of at least three independent experiments, each in triplicates. Error bars indicate S.E.M. The magnitude of the increase in the percentage of *GFP*$^+$ cells by NU7441 treatment is shown above each column. Significance was analyzed by two-tailed Student's paired t-test between "DMSO" and "NU7441" and indicated by * for P<0.05, ** for P<0.01 and n.s for not significant.

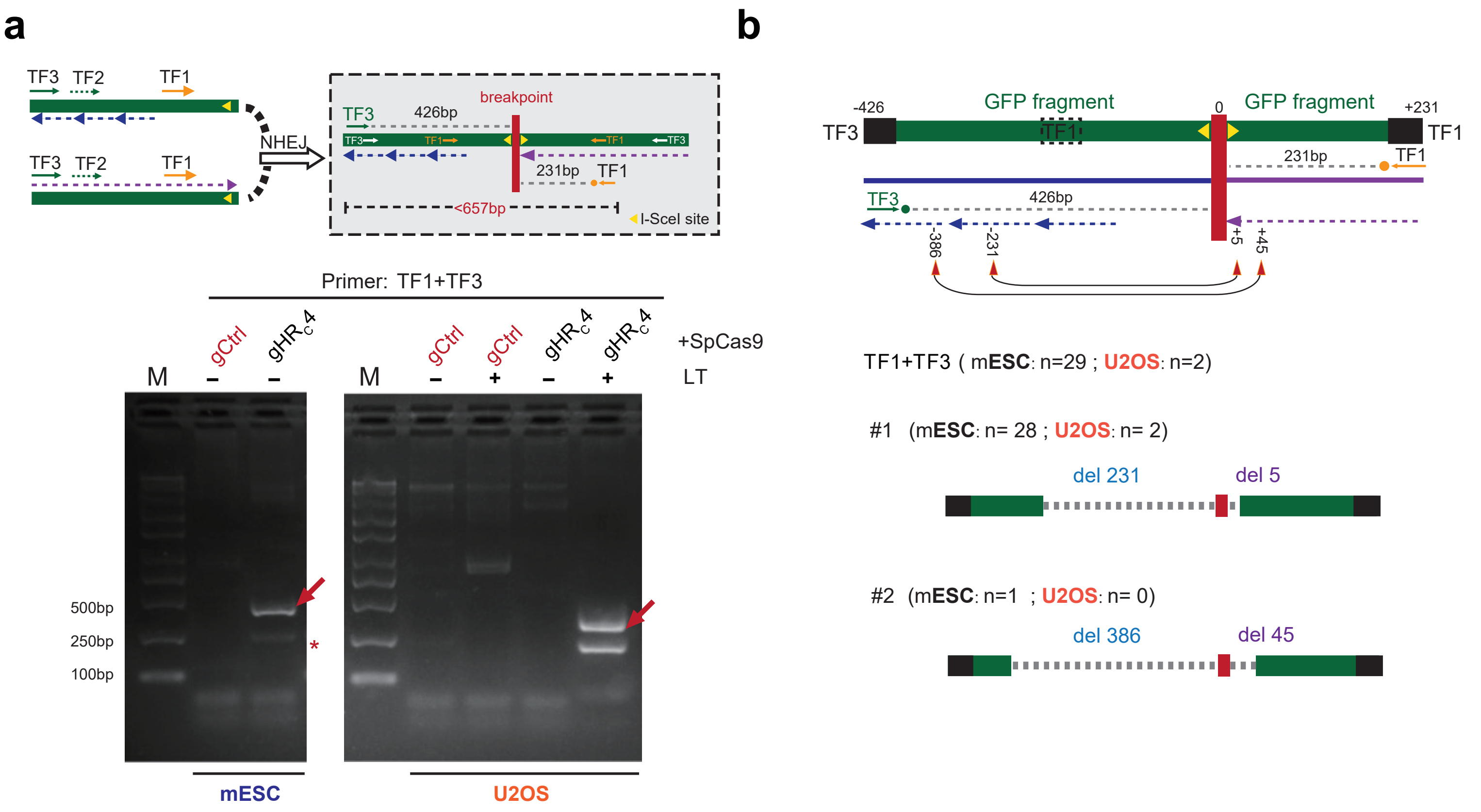

Fig S8 Analysis of palindromic sister chromatid ligation with the primer pair TF1 and TF3. **a** PCR detection of palindromic sister chromatid ligation. HDR reporter mESC and HDR reporter U2OS cells were transfected with SpCas9-gHRC4 expression plasmids or empty vector as indicated. HDR reporter U2OS cells were also co-transfected with SV40 *LT* expression plasmids or control vector. At 2 d post-transfection, gDNA was isolated, and PCR was performed with the primer pair TF1 and TF3 and analyzed by electrophoresis. The distance between TF1 or TF3 and the break point was indicated. M: 5kb DNA ladder. *: non-specific PCR bands. **b** Repair junction of palindromic sister chromatid NHEJ products. The PCR products from (A) were subcloned and detected by Sanger sequencing, and the sequencing data were pooled from two independent experiments. Only two types of products (#1 and #2) were detected with the size and position of deletion (del) and insertion (ins) as indicated, and the number of products from mESC and U2OS cells were also shown.

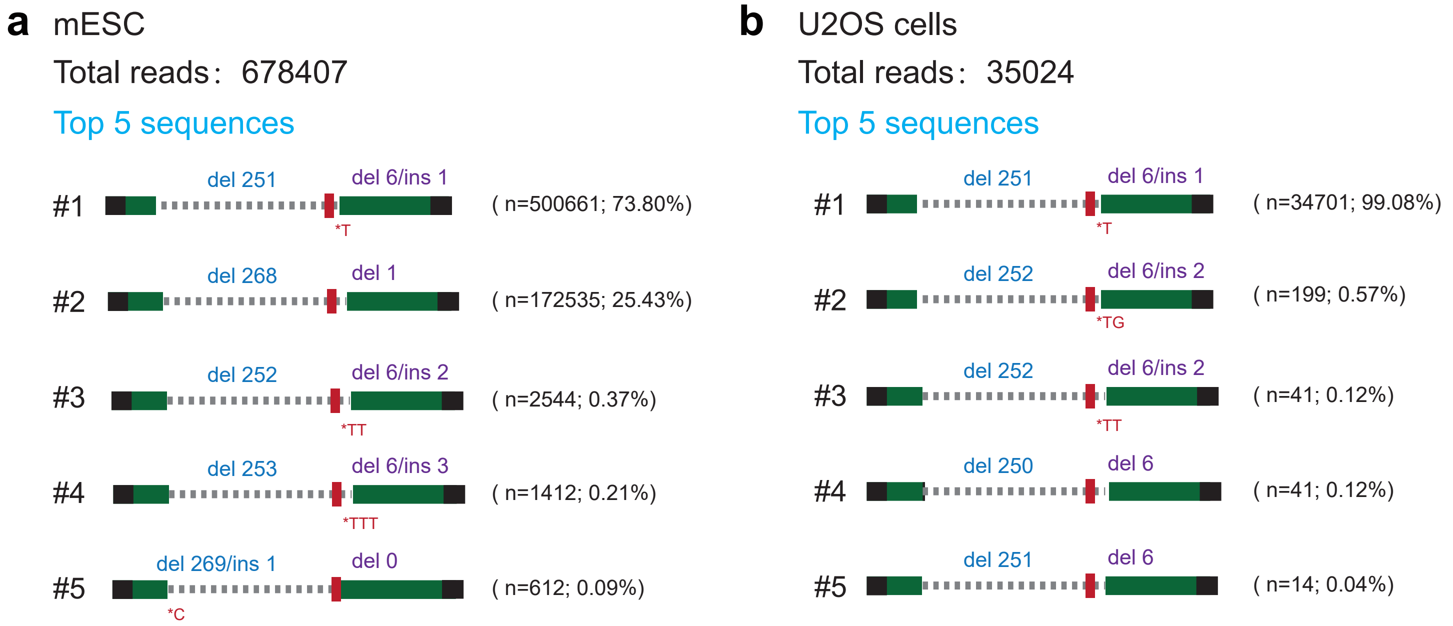

Fig S9 Junctions of palindromic sister chromatid NHEJ products in mESC (**a**) and U2OS cells (**b**) by deep sequencing analysis. After target amplification by PCR with the primer pair TF1 and TF2, amplicons of palindromic sister chromatid NHEJ products were analyzed by deep sequencing. Total reads and top 5 structures of repair junctions are shown along with their respective sequencing reads and frequencies. *T, *C, *TT, *TG and *TTT indicate nucleotides and positions inserted.
