# Supplementary material for: Target residence of Cas9-sgRNA influences DNA double-strand break repair pathway choices in CRISPR/Cas9 genome editing
Source: Genome Biol. 2022 Aug 1;23:165. doi: 10.1186/s13059-022-02736-5 (PMC9341079; doi:10.1186/s13059-022-02736-5)
Supplement: Supplementary file 2 — Additional file 2: Table S1. Junction sequences of sister chromatid fusion by Sanger sequencing. [file 13059_2022_2736_MOESM2_ESM.pdf]

Table S1. Junction sequences of sister chromatid fusion by Sanger sequencing

---

TF1+TF2

ES #1 (n=11)

GGCCACAAGTTCAGCGTGTCCGGCGAGGGCGAGGGCGATGCCACCTACGGCAAGCTG
ACCCTGAAGTTCATCTGCACCACCGGCAAGCTGCCCGTGCCCTGGCCCACCCTCGTGA
CCACCCTGACCTACGGCGTGCAGTGCTTCAGCCGCTACCCCGACCACATGAAGCAGCA
CGACTTCTTCAAGTCCGCCATGCCCGAAGGCTACGTCCAGGAGCGCACCATCTTCTTC
AAGGACGACGGCAACTACAAGACCCGCGCCGAGGTGAAGTTCGAGGGCGACACCCT
GGTGAACCGCATCGAGCTGAAGGGCATCGTAGGGATAACAGGGTAATCA^TGATTA**T**C
CCTGTTATCCCTACGATGCCCTTCAGCTCGATGCGGTTCACCAGGGTGTCGCCCTCGAA
CTTCACCTCGGCGCGGGTCTTGTAGTTGCCGTCGTCCTTGAAGAAGATGGTGCGCTCC
TGGACGTAGCCTTCGGGCATGGCGGACTTGAAGAAGTCGTGCTGCTTCGATGTGGTCG
GGGTAGCGGCTGAAGCACTGCACGCCGTAGGTCAGGGTGGTCACGAGGGT

ES #2 (n=6)

GGCCACAAGTTCAGCGTGTCCGGCGAGGGCGAGGGCGATGCCACCTACGGCAAGCTG
ACCCTGAAGTTCATCTGCACCACCGGCAAGCTGCCCGTGCCCTGGCCCACCCTCGTGA
CCACCCTGACCTACGGCGTGCAGTGCTTCAGCCGCTACCCCGACCACATGAAGCAGCA
CGACTTCTTCAAGTCCGCCATGCCCGAAGGCTACGTCCAGGAGCGCACCATCTTCTTC
AAGGACGACGGCAACTACAAGACCCGCGCCGAGGTGAAGTTCGAGGGCGACACCCT
GGTGAACCGCATCGAGCTGAAGGGCATCGTAGGGATAACAGGGTAATCA^TGATTA**(T)**
CCCTGTTATCCCTACGATGCCCTTCAGCTCGATGCGGTTCACCAGGGTGTCGCCCTCGA
ACTTCACCTCGGCGCGGGTCTTGTAGTTGCCGTCGTCCTTGAAGAAGATGGTGCGCTC
CTGGACGTAGCCTTCGGGCATGGCGGACTTGAAGAAGTCGTGCTGCTTCGATGTGGTC
GGGGTAGCGGCTGAAGCACTGCACGCCGTAGGTCAGGGTGGTCACGAGGGT

U2OS #1 (n=23)

GGCCACAAGTTCAGCGTGTCCGGCGAGGGCGAGGGCGATGCCACCTACGGCAAGCTG
ACCCTGAAGTTCATCTGCACCACCGGCAAGCTGCCCGTGCCCTGGCCCACCCTCGTGA
CCACCCTGACCTACGGCGTGCAGTGCTTCAGCCGCTACCCCGACCACATGAAGCAGCA
CGACTTCTTCAAGTCCGCCATGCCCGAAGGCTACGTCCAGGAGCGCACCATCTTCTTC
AAGGACGACGGCAACTACAAGACCCGCGCCGAGGTGAAGTTCGAGGGCGACACCCT
GGTGAACCGCATCGAGCTGAAGGGCATCGTAGGGATAACAGGGTAATCA^TGATTA**T**C
CCTGTTATCCCTACGATGCCCTTCAGCTCGATGCGGTTCACCAGGGTGTCGCCCTCGAA
CTTCACCTCGGCGCGGGTCTTGTAGTTGCCGTCGTCCTTGAAGAAGATGGTGCGCTCC
TGGACGTAGCCTTCGGGCATGGCGGACTTGAAGAAGTCGTGCTGCTTCGATGTGGTCG
GGGTAGCGGCTGAAGCACTGCACGCCGTAGGTCAGGGTGGTCACGAGGGT

TF1+TF3

ES #1 (n=28)

TACCGGTCGCCACCATGGTGAGCAAGGGCGAGGAGCGGTATGTTCACCGGGGTGGTG
CCCATCCTGGTCGAGCTGGACGGCGACGTAAACGGCCACAAGTTCAGCGTGTCCGGC

GAGGGCGAGGGCGATGCCACCTACGGCAAGCTGACCCTGAAGTTCATCTGCACCACC
GGCAAGCTGCCCGTGCCCTGGCCCACCCTCGTGACCACCCTGACCTACGGCGTGCAGT
GCTTCAGCCGCTACCCCGACCACATGAAGCAGCACGACTTCTTCAAGTCCGCCATGCC
CGAAGGCTACGTCCAGGAGCGCACCATCTTCTTCAAGGACGACGGCAACTACAAGAC
CCGCGCCGAGGTGAAGTTCGAGGGCGACACCCTGGTGAACCGCATCGAGCTGAAGGG
CATCGTAGGGATAACAGGGTAATCA^TGATTACCCTGTTATCCCTACGATGCCCTTCAGC
TCGATGCGGTTCACCAGGGTGTCGCCCTCGAACTTCACCTCGGCGCGGGTCTTGTAGT
TGCCGTCGTCCTTGAAGAAGATGGTGCGCTCCTGGACGTAGCCTTCGGGCATGGCGGA
CTTGAAGAAGTCGTGCTGCTTCGATGTGGTCGGGGTAGCGGCTGAAGCACTGCACGCC
GTAGGTCAGGGTGGTCACGAGGGT

ES #2(n=1)

TACCGGTCGCCACCATGGTGAGCAAGGGCGAGGAGCGGTATGTTCACCGGGGTGGTG
CCCATCCTGGTCGAGCTGGACGGCGACGTAAACGGCCACAAGTTCAGCGTGTCCGGC
GAGGGCGAGGGCGATGCCACCTACGGCAAGCTGACCCTGAAGTTCATCTGCACCACC
GGCAAGCTGCCCGTGCCCTGGCCCACCCTCGTGACCACCCTGACCTACGGCGTGCAGT
GCTTCAGCCGCTACCCCGACCACATGAAGCAGCACGACTTCTTCAAGTCCGCCATGCC
CGAAGGCTACGTCCAGGAGCGCACCATCTTCTTCAAGGACGACGGCAACTACAAGAC
CCGCGCCGAGGTGAAGTTCGAGGGCGACACCCTGGTGAACCGCATCGAGCTGAAGGG
CATCGTAGGGATAACAGGGTAATCA^TGATTACCCTGTTATCCCTACGATGCCCTTCAGC
TCGATGCGGTTCACCAGGGTGTCGCCCTCGAACTTCACCTCGGCGCGGGTCTTGTAGT
TGCCGTCGTCCTTGAAGAAGATGGTGCGCTCCTGGACGTAGCCTTCGGGCATGGCGGA
CTTGAAGAAGTCGTGCTGCTTCGATGTGGTCGGGGTAGCGGCTGAAGCACTGCACGCC
GTAGGTCAGGGTGGTCACGAGGGT

U2OS #1 (n=2)

TACCGGTCGCCACCATGGTGAGCAAGGGCGAGGAGCGGTATGTTCACCGGGGTGGTG
CCCATCCTGGTCGAGCTGGACGGCGACGTAAACGGCCACAAGTTCAGCGTGTCCGGC
GAGGGCGAGGGCGATGCCACCTACGGCAAGCTGACCCTGAAGTTCATCTGCACCACC
GGCAAGCTGCCCGTGCCCTGGCCCACCCTCGTGACCACCCTGACCTACGGCGTGCAGT
GCTTCAGCCGCTACCCCGACCACATGAAGCAGCACGACTTCTTCAAGTCCGCCATGCC
CGAAGGCTACGTCCAGGAGCGCACCATCTTCTTCAAGGACGACGGCAACTACAAGAC
CCGCGCCGAGGTGAAGTTCGAGGGCGACACCCTGGTGAACCGCATCGAGCTGAAGGG
CATCGTAGGGATAACAGGGTAATCA^TGATTACCCTGTTATCCCTACGATGCCCTTCAGC
TCGATGCGGTTCACCAGGGTGTCGCCCTCGAACTTCACCTCGGCGCGGGTCTTGTAGT
TGCCGTCGTCCTTGAAGAAGATGGTGCGCTCCTGGACGTAGCCTTCGGGCATGGCGGA
CTTGAAGAAGTCGTGCTGCTTCGATGTGGTCGGGGTAGCGGCTGAAGCACTGCACGCC
GTAGGTCAGGGTGGTCACGAGGGT

Note: The numbers in parentheses denotes the sample numbers for product type #1 and #2. Red reversed V represent the breakpoints generated by *Sp*Cas9-gHRc4. The position and direction of primers are indicated on the sequences. TF1 primer was underlined by Red line, TF2 primer was underlined by Black line and TF3 primer was underlined by Blue line. The grey shaded area represents the deleted sequences that were initially

expected in the palindromic sister chromatid NHEJ products. The inverted *GFP* sequences are denoted in green. Red bold letter denotes an inserted base. Microhomology at the junctions is underlined.
