# Supplementary material for: Target residence of Cas9-sgRNA influences DNA double-strand break repair pathway choices in CRISPR/Cas9 genome editing
Source: Genome Biol. 2022 Aug 1;23:165. doi: 10.1186/s13059-022-02736-5 (PMC9341079; doi:10.1186/s13059-022-02736-5)
Supplement: Supplementary file 5 — Additional file 5: Table S4. List of sgRNAs used in this study. [file 13059_2022_2736_MOESM5_ESM.pdf]

Table S4. List of sgRNAs used in this study

sgRNA	Sequence (5'-3')	PAM	RNA-bound Strand	Length (bp)
**For *Sp*Cas9**				
gEJ$_c$5	TGGATTACCCTGTTATCCCT	AGG	C	20
gEJ$_w$3	AGGGATAACAGGGTAATCCA	TGG	W	20
gEJ$_w$4	GGATGGATCCTAGGGATAAC	AGG	W	20
gEJ$_w$6	TGGATTACCCTGTTATCCCT	AGG	W	20
gEJ$_w$5	CATGTGAAGGATGGATCCTA	GGG	W	20
gEJ$_w$7	CTTGCTGATCATGTGAAGGA	TGG	W	20
gEJ$_c$5-C2A	TGGATTACCCTGTTATCCAT	AGG	C	20
gEJ$_c$5-T15A	TGGATAACCCTGTTATCCCT	AGG	C	20
gEJ$_c$5-16nt	TTACCCTGTTATCCCT	AGG	C	16
gEJ$_w$7-A1T	CTTGCTGATCATGTGAAGGT	TGG	W	20
gEJ$_w$7-A4C	CTTGCTGATCATGTGACGGA	TGG	W	20
gEJ$_w$7-T15A	CTTGCAGATCATGTGAAGGA	TGG	W	20
gI-SceI site	TGGCGGCggtTAGGGATAAC	AGG	W	20
gHR$_c$3	TAGGGATAACAGGGTAATCA	AGG	C	20
gHR$_c$4	GATAACAGGGTAATCAAGG	AGG	C	19
gHR$_c$1	TCGAGCTGAAGGGCATCGTA	GGG	C	20
gHR$_c$2	AGGGCATCGTAGGGATAACA	GGG	C	20
gHR$_c$5	AACAGGGTAATCAAGGAGGA	CGG	C	20
gHR$_c$4-G1C	GATAACAGGGTAATCAAGC	AGG	C	19
gHR$_c$4-G2C	GATAACAGGGTAATCAACG	AGG	C	19
gHR$_c$4-17nt	TAACAGGGTAATCAAGG	AGG	C	17
gHR$_c$2-A1T	AGGGCATCGTAGGGATAACT	GGG	C	20
gHR$_c$2-C2A	AGGGCATCGTAGGGATAAAA	GGG	C	20
gHR$_c$2-17nt	GCATCGTAGGGATAACA	GGG	C	17
gG$_c$1	GGCCACAAGTTCAGCGTGTC	CGG	C	20
gG$_w$1	GCGCTCCTGGACGTAGCCTT	CGG	W	20
gG$_c$4	CATGCCCGAAGGCTACGTCC	AGG	C	20
gG$_c$5	ACCATCTTCTTCAAGGACGA	CGG	C	20

gG_c7	GAAGTTCGAGGGCGACACCC	TGG	C	20
gG_w5	CAGCTCGATGCGGTTCACC	AGG	W	19
gG_c9	GCTGAAGGGCATCGACTTCA	AGG	C	20
gG_c10	GAAGGGCATCGACTTCAAGG	AGG	C	20
gG_w2	TGAAGAAGATGGTGCGCTCC	TGG	W	20
gG_w6	CGATGCCCTTCAGCTCGATG	CGG	W	20
gG_c12	AGGAGGACGGCAACATCCTG	GGG	C	20
gG_w7	TGTACTCCAGCTTGTGCCCC	AGG	W	20
gG_c14	ATGGCCGACAAGCAGAAGAA	CGG	C	20
gG_c15	AAGCAGAAGAACGGCATCA	AGG	C	19
gG_w10	TGCCGTCCTCGATGTTGTGG	CGG	W	20
gG_c17	ATCCGCCACAACATCGAGGA	CGG	C	20
gR1	CCAACCCCTAGACAGAGCAT	TGG	C	20
gR2	CAGACTTCTAAGATCAGGAA	AGG	W	20
gR3	GTGTATGTAACTAATCTGTC	TGG	W	20
gR4	ACTGAGCTATTAACCATTAA	TGG	C	20
gR4-A1C	ACTGAGCTATTAACCATTAC	TGG	C	20
gR4-A16T	ACTGTGCTATTAACCATTAA	TGG	C	20
gC1	TTCCCAGGCATCACCAGTCT	TGG	C	20
gC4	GCCAGGAAGAGTCCCAAGAC	TGG	W	20
gC2	AGCAGTGGCCAGGTACAGCT	CGG	W	20
gC3	CAGACAAGCGAGCAGTGGCC	AGG	W	20
gC4-C1T	GCCAGGAAGAGTCCCAAGAT	TGG	W	20
gC4-G16C	GCCACGAAGAGTCCCAAGAC	TGG	W	20
gPnpla3	GGGATTAGGCCAGAGAAGAG	GGG	W	20
gMertk	CGCCAGGAACTCACACTTCA	TGG	W	20
gDNA-PKcs-1	TGTTGGCTACTGCAGCTGC	AGG	C	19
gDNA-PKcs-1a	CAGACCGCTGCAGTGCTGC	CGG	C	19
gKu80-1	TGTGGATGTGGGGGTTGCCA	TGG	C	20
gKu80-2	TCAATTGGAGATTCTTCACC	AGG	W	20
**For *Sa*Cas9**				
gSa-Gc1	TCAGATCTCGAGCTCAAGCT	TCGAAT	C	20
gSa-Gw1	TCGAAGCTTGAGCTCGAGAT	CTGAGT	W	20

gSa-Gw2	AGGATGGATCCTAGGGATAA	CAGGGT	W	20
gSa-Gw3	TCATGTGAAGGATGGATCCT	AGGGAT	W	20
gSa-Gw4	CCTTGCTGATCATGTGAAGG	ATGGAT	W	20
gSa-Gw5	TCGCCCTTGCTGATCATGTG	AAGGAT	W	20
