# Supplementary material for: Target residence of Cas9-sgRNA influences DNA double-strand break repair pathway choices in CRISPR/Cas9 genome editing
Source: Genome Biol. 2022 Aug 1;23:165. doi: 10.1186/s13059-022-02736-5 (PMC9341079; doi:10.1186/s13059-022-02736-5)
Supplement: Supplementary file 6 — Additional file 6: Table S5. List of PCR primers used in this study. [file 13059_2022_2736_MOESM6_ESM.pdf]

**Table S5. List of PCR primers used in this study**

| PCR primers | Sequence (5'-3') |
|---|---|
| mCola1-F | CCACTGATTGACACGTTGATTG |
| mCola1-R | AGTCACACCCTTTGTCTGGTAT |
| | |
| mRosa26-gR4-F | ACAACAATAGATGTATTGAG |
| mRosa26-gR4-R | ACCTTTTACAGATGTGTACA |
| | |
| | |
| mKu80-F | GGCCTTTGTTTCTAGGCAG |
| mKu80-R | TGGACAAACATAGTCATCAC |
| | |
| mDNA-PKcs-F | TAGAGTGCTGAGACGCGCAT |
| mDNA-PKcs-R | GCACTCCTGCCCCAGACTA |
| | |
| TF1 | ACCCTCGTGACCACCCTGACCTACGGC |
| TF2 | GGCCACAAGTTCAGCGTGTCCGGCGAG |
| TF3 | TACCGGTCGCCACCATGGTG |
| | |
| **OFF-Target site primers** | |
| ***Sp*Cas9+gHRc1** | |
| On-Target-P1-F | CTAGCGCTACCGGTCGCCAC |
| On-Target-P1-R | TGATCTAGAGTCGCGGCCGC |
| On-Target-P2-F | CGAAGGCTACGTCCAGGAGCGCACCAT |
| On-Target-P2-R | TGTGGCGGATCTTGAAGTTCACCTTGA |
| OT1-F | GCAAAACCTGGAGTAATGTAAGTG |
| OT1-R | CCCTCTTCTATGTGACTGATAGT |
| OT2-F | CCGCTCTGGATTTTCTGTAGC |
| OT2-R | GGCCAATGGGGTTTGGTGCT |
| OT3-F | GACCTACCCAAAACTCTACATAG |
| OT3-R | TCCTGTTACTGTTCTCGTTCTC |
| OT4-F | ATGGACCACATGTATGCAGTCC |
| OT4-R | CCACCTGCCAATGGTTTTAGAC |
| OT5-F | GTCTTATGCCTACAGCAACCAT |
| OT5-R | GTAAAGAGCCTATTGGCTGAATGA |
| OT6-F | GCACTCATTCCCTAGTATAAAATCTG |
| OT6-R | CAAGCACTTGGGAGGTAGAAG |
| | |
| ***Sp*Cas9+gHRc2** | |
| On-Target-P1-F | CTAGCGCTACCGGTCGCCAC |
| On-Target-P1-R | TGATCTAGAGTCGCGGCCGC |
| On-Target-P2-F | CGAAGGCTACGTCCAGGAGCGCACCAT |
| On-Target-P2-R | TGTGGCGGATCTTGAAGTTCACCTTGA |
| OT1-F | GATCTCCATAGGAGGAAAGAAGT |
| OT1-R | GCTTCATTTGTTAACCAGAGATGG |
| OT2-F | GTCCACTGAGGCATTTTCCCA |
| OT2-R | TCCTCACAGCTACCTGATATTCAA |
| OT3-F | CCCTCTGATCAATTAGATGATGGA |

| | |
|---|---|
| OT3-R | CATTGACAACACATGTTACATTTTGTGC |
| OT4-F | CCTGATGCTGATGGCACTGA |
| OT4-R | GGGTAGGGTGATTCAATCAATAATG |
| OT5-F | CTGTGCCACCTGCTTTCATTAACT |
| OT5-R | CATTTATCTTGAACCATTTGGCCAC |
| OT6-F | CATAGAGCTTTGAACAGTATCTGTG |
| OT6-R | CCAAGTCAAAGGTTTAGGATTTCTG |
| | |
| ***Sp*Cas9+gPnpla3** | |
| On-Target-F | GCATCTCACTTACACTGCTCAC |
| On-Target-R | CAAGACTCAGATCCCAGCTC |
| OT1-F | TAGAAGGGTCCCTAGTCATTCC |
| OT1-R | GTCACATTGTTCTCCTCAGATAC |
| OT2-F | ATTTTAATGTGTTTGTGGGGCAAGAAAC |
| OT2-R | GGAGATTTATGTTTGTTTTGTCATCTGA |
| OT3-F | CTGGCTATACTCTCCCACATAT |
| OT3-R | TTCAGCCAAGTCAGCAGAGC |
| OT4-F | TCTGTGACATCATCCCTTACCC |
| OT4-R | AGGAGATATGAGAAGGCTATAGC |
| OT5-F | CTTGCTATGATCCATCCAGACTC |
| OT5-R | CTCCTTCCATCAGAAGATAGGA |
| OT6-F | CATACTTTCCCTAAGAATCTTGATGC |
| OT6-R | AGCTACTTGTAGACTCTGTAGC |
| OT7-F | GTCTGCCTCTGTTCCCTCTA |
| OT7-R | GCATGTAGAAAGATCACCTGTTC |
| | |
| ***Sp*Cas9+gMertk** | |
| On-Target-F | AAAGCATCTGCGCAGCACTGC |
| On-Target-R | CTTCCACATTGATCGTCACACC |
| OT1-F | CAAGTCTCTAGAATTGGAAGCTAC |
| OT1-R | GTGACATCCAGAAACACTCACC |
| OT2-F | TCAGATTAAAGACACCACTCTCCT |
| OT2-R | CACTGAACTCTAACCTTTCTATGG |
| OT3-F | TGACCCACGTGTGGAAGCTG |
| OT3-R | TTCAAAGCAAACAATGTGGAGAAAGTA |
| OT4-F | CTAATGTACTGTAGAATGTGTGCC |
| OT4-R | CTCAGCCTTCTCTAGGACTG |
| OT5-F | GACTATGTGGGTGTGAACAGG |
| OT5-R | AAACAACTTCACTTGTGTAAAATTTCC |
| OT6-F | ACATATAACACACAGACAGCATACAC |
| OT6-R | GAAAGCACAGCAAGGAAGAGAC |
| | |
| DyLight680-HDR-F | ATGGTGAGCAAGGGCGAGGAGCTG |
| DyLight680-HDR-R | CAGGTAGTGGTTGTCGGGCAGCAG |
| DyLight800-NHEJ-F | AGAGGCTGGGAAGGGGTGGGTCCG |
| DyLight800-NHEJ-R | ACTTCACCTCGGCGCGGGTCTTGT |
